# Toxoplasmosis in Iran: A guide for general physicians working in the Iranian health network setting: A systematic review

**Published:** 2016

**Authors:** Seyed Mohammad Alavi, Leila Alavi

**Affiliations:** 1 Health Research Institute, Infectious and Tropical Disease Research Center, Joundishapur University of Medical Sciences, Ahvaz, Iran; 2 Food and Drug Deputy, Jundishapur University of Medical Sciences, Ahvaz, Iran

**Keywords:** Toxoplasma infection, *T. gondii*, Epidemiology, Health center, Iran

## Abstract

**Background:**

Human toxoplasmosis is an important zoonotic infection worldwide which is caused by the intracellular parasite *Toxoplasma gondii *(T.gondii). The aim of this study was to review briefly the general aspects of toxoplasma infection in in Iranian health system network.

**Methods:**

We searched published toxoplasmosis related articles in English databases including Science Direct, Pub Med, Scopus, Google Scholar, Magiran, Iran Medex, Iran Doc and Scientific Information Database (SID) for toxoplasmosis.

**Results:**

Out of 1267 articles from the English and Persian databases search, 40 articles were suitable with our research objectives and so were selected for the study. It is estimated that at least a third of the world human population is infected with T.gondii, suggesting it as one of the most common parasitic infections through the world. Maternal infection during pregnancy may affect dangerous outcome for the fetus, or even cause intrauterine death. Reactivation of a previous infection in immunocompromised patient such as drug induced, AIDS and organ transplantation can cause life-threating central nervous system infection. Ocular toxoplasmosis is one of the most important causes of blindness, especially in individuals with a deficient immune system.

**Conclusion:**

According to the increasing burden of toxoplasmosis on human health, the findings of this study highlight the appropriate preventive measures, diagnosis, and management of this disease.

*Toxoplasma gondii (T. gondii*) is an intracellular parasite which infects both humans and animals and causes one of the most common zoonotic diseases in the world (1). It is estimated that at least a third of the world human population is infected with T.gondii, suggesting it as one of the most common parasitic infections worldwide. (2). Previous studies revealed that the seroprevalence rate of toxoplasma infection varies between 30% and 60% in both the developed and the developing countries (3). The prevalence of toxoplasmosis varies according to geographic regions with respect to climates, environmental conditions, lifestyles, health facilities and hygiene status (4). In a study conducted in France, up to 43% of the French population were infected, due to their lifestyle, eating habits and exposure to cats. The two types of infection are described as congenital and acquired form (5). Toxoplasmosis-associated illnesses in immunocompetent individuals, include regional lymphadenopathy (6), neuropsychologic disorders (7) ocular disease and congenital neurologic, whereas, in immunosuppressed individuals toxoplasmosis presents as encephalitis or disseminated disease. 

Overall the total prevalence of 40% toxoplasmosis infection in the general population of Iran has been reported by Daryani et al., (8). This issue suggests the importance of these infective organisms to the public health. Therefore, pregnant women, children with congenital infection and immunocompromised persons such as HIV infected patients or organ transplanted patients are the most important individuals who are at risk of infection or reactivation of latent infection (9). General physicians as well as other health care workers (HCWs) who are concerned with public health should not overlook the dangers of toxoplasmosis in health care settings. The aim of this article was to review the current knowledge, epidemiology, diagnosis, treatment and prevention of toxoplasmosis among high risk persons attending health care workplace.

 Out of 1267 articles from the English and Persian databases search, 40 articles were suitable with our research objectives and were selected for the study. Table 1 shows the results of the literature search about Iranian health system, the status of epidemiology of toxoplasmosis in the world and Iran, diagnostic methods available in health setting, treatment and useful recommendation for prevention according to the lifestyle of the people living in the region of study.

**Table 1:** Characteristics of selected articles from literature search

**Subject **	**Year **	**Location site**	**References **
Toxoplasmosis; pathogenesis	2011	USA	Subauste et al.
Toxoplasmosis; prevalence	2000 2014	Australia Iran	Tenter et al Daryani et al
Clinical presentation; schizophrenia	2003,2012, 2009	Czech Iran	Fleger et al.,Fond et al., Hamidinejat et al.
Toxoplasmosis ;review	2006,2004 2012	USA Iran	Dodds et al., Montaya et al. Seadatnia et al.
Toxoplasmosis; treatment	2009 2010 2001 2002	Iran Peru France USA	Alavi et al. Nunura et al. Deroin et al. Camps et al.
Health system network	2004,2013	Iran	Asadi et al., Alavi et al
Neglected diseases	2014	USA	Jones et al.
Toxoplasmosis; transmission	2014, 2004 2014	Czech USA Iran	Fleger et al. Fayer et al. Ebrahimi et al.
Infection during pregnancy	2014 2014,2013 2013 2008	India Iran Iran USA	Chaudry et al. Davami et al., Elahian et al., Asgari et al. Montaya et al
Toxoplasmosis; traveling	2014	USA	Sepulveda Arias et al.
Textbook; infectious diseases	2010	USA	Mandel et al.
Congenital infection	2006 2009	USA France	Roman et al. Garcia-Meric et al
Clinical presentation; fever of unknown origin	2013	India	Abhilibash et al
Clinical presentation; disseminated infection	2010 2013	Peru Italy	Nunura et al Cumo et al
Infection among immunocompromised host	2013	Iran	Alavi et al
Ocular toxoplasmosis	2014 2004	Iran Iran	Rahimi et al. Soheilian et al
Clinical presentation; lymphadenitis	2014	Saudi Arabia	Bilal et al
Toxoplasmosis; diagnosis	2007 2014	France Iran	Leal et al Sarkari et al.
Toxoplasmosis; prevention	2002, 2008 2005 2006	France USA Brazil	Peloux et al., Deroin et al. Kerayetz et al de Moura et al.

**Health Care Network in Iran:** Iranian Ministry of ealth and Medical Education is in charge of provision of healthcare services through its network of medical sciences universities including health centers in the country. An elaborate system of health network has been established which has ensured provision of primary health services to the vast majority of public (10, 11). 

**Primary Health Center:** The Iranian health care network includes first line health unit called in Iran" Khaneh-e-Behdasht", rural health centers, urban health centers and tertiary hospitals. Khaneh-e-Behdasht is the fundamental unit of the public health system providing primary health care (PHC) to people living in rural areas. Since 1984, the activities of the health system have resulted in a dramatic decrease in the burden of common and endemic infectious diseases (11). The health centers provide appropriate health services throughout Iran, from remote mountain areas to inner urban areas in the country’s capital (10, 11).

**Epidemiology of toxoplasmosis:** Cats are the definitive host for T.gondii where the parasite can complete its sexual cycle. Cats usually shed the oocyst in their feces for up to 2 weeks after a new infection. Oocystes are the environmentally-resistant form of organism. Sporulation is needed for oocysts to become infectious and this happens within 1–5 days in the environment. Sporulated oocysts can remain infective in a humid environment for a year or longer. When ingested by humans or susceptible animal, the parasite becomes a tissue-infective form in the intestine, enters the blood system and migrates via the blood to other body organs (12, 13).

**Route of transmission:** Humans are most commonly infected accidentally by either ingestion of contaminated food, drinking contaminated water, or soil having oocysts from a cat’s feces, or eating raw meat containing tissue cysts (14). Infection in humans also occurs congenitally from mother who acquired her infection during gestation to her child through transplacental transmission (15). Less common ways of toxoplasma transmission are by transplantation of an infected organ, or transfusion of infected blood, or transmission by accidental needlesticks, or by hemodialysis, or through sexual transmission (13, 6-19). 

The transmission of toxoplasmosis infection via organ transplantation may result from a seropositive donor to a seronegative recipient. In bone marrow transplant recipients, toxoplasmosis is always a result of reactivation of a latent infection rather than from the infected transplanted organ (20).

**Prevalence in the world:** The seroprevalence of *T.gondii *varies among different populations from different geographic areas and even within a given population. These differences depend on various factors such as eating habits and general health. A decrease in seroprevalence over the past few decades has been reported worldwide (1, 2). Serological surveys have demonstrated that exposure to T. gondii even in the developed countries is high, from 30% in the United States to 50-80% in Europe (21).

**Prevalence in Iran: ** In a recently published systematic review study and meta-analysis has reported that the overall toxoplasma seroprevalence rate among the general population in Iran was approximately 40%; there was no significant difference between males and females. An increasing trend of seroprevalence by age was suggested. In addition, there was high seroprevalence in those who have a direct contact with cats, consume uncooked meat and raw fruits or vegetable use, and work in farms, such as a housewife with low level of education, and live in rural areas(8).

T. gondii seropositivity among a sample of young Iranian women who presented for pre-marital laboratory test using ELISA was 15% (13% for IgG and 2% for IgM). Seropositivity for T. gondii IgG differed according to age groups. IgM seropositivity showed the highest rate among women aged < 20 years. They concluded that appropriate educational programs to improve knowledge in young women who are at the risk of infection during gestation should be implemented (22). 

**Clinical Manifestations:** *Toxoplasmosis *has a wide spectrum of clinical diseases, from asymptomatic infection in immunocompetent person to a life-threatening illness in immunodeficient patient. Toxoplasmosis is classified as: asymptomatic infection, ocular disease, infection in pregnancy, acquired or reactivated infection in the immunocompromised patient, and congenital infection. 

General physicians and health care workers (HCWs) involved in Iranian health network regarding their work duties in providing basic health services are interested in investigating toxoplasma infection in immunodeficient patients, pregnant women and children with ocular infection. Therefore, in summary in this review we seek to address these three major issues.

**Immunocompromised patients:** In contrast, toxoplasmosis is usually benign in normal individuals, the infection in immunocompromised individuals is almost always dangerous and malignant because of its devastating consequences, and therefore there is a need of rapid diagnosis and management of toxoplasmosis in these patients (9, 18, 20).

Immunocompromised patients included those individuals with hematologic malignancies, organ transplantation, HIV/ AIDS, and those under treatment of immunosuppressive agents such as corticosteroids, anti-TNF drugs and cytotoxic drugs. In patients with immunodeficiency status, active disease, include encephalitis, pneumonitis, and myocarditis. Pneumonitis is usually under recognized and neglected (20). Fever of unknown origin may be the only clinical presentation of toxoplasmosis in the early stages of T. gondii infection (23). Disseminated infection may be the other form of infection that is reported frequently among immunocompromised patients, although rare cases of this form have been found in immunocompetent persons (20, 24). Mortality is high and up to 100% if the infection is not diagnosed and appropriately treated. Although clinical manifestations in organ transplant recipient are similar to other immunocompromised patients, additional considerations should be provided to organ transplant recipient and AIDS patients (20, 25). Iran, in the light of HIV/AIDS epidemic is a country in which HIV is concentrated in intravenous drug users (IVDUs). In a study conducted by Alavi et al., in Ahvaz, southwest Iran, the frequency of toxoplasma-IgG in HIV positive and HIV negative IVDUs was 73.8% and 81%, respectively. Based on their results, prevalence of toxoplasmosis infection in the illicit drug users with HIV positive or negative is equal.

It is well shown that in hemodialysis patients, the humoral and cellular immune systems are suppressed and the number of circulating T cell is reduced. Hemodialysis cannot be returned to impair immune status. Thus, immunosuppression causes high prevalence of latent infections such as toxoplasmosis (16). Ebrahimzadeh et al. in their case-control study reported a high prevalence of toxoplasmosis among hemodialysis patients. Other findings indicate that 21 out of 37 patients (58.3%) in the case group were positive for anti-t*oxoplasma gondi* IgG in case group while in control group only 11 (29.7%) individuals were positive that had statistically significant difference(p <0.05 ) (17).

**Pregnant women:** Like other persons with normal immune system, acute *toxoplasma *infection in pregnant women is usually asymptomatic. The most common clinical manifestation of recent infection in pregnancy is regional lymphadenopathy (20).Less commonly, pregnant women like other immunocompetent individuals may be clinically presented with atypical features such as FUO, tonsillitis, pneumonia, polyneuritis and polymyositis (23, 24, 26). The risk of infection from mother to the fetus does not relate with clinical presentation of infection in the mother during gestation. Transmission to the fetus is limited to acquiring the infection during gestation (27).

 Maternal infection before becoming pregnant does not result in transmitting the infection to the fetus. Despite the fact that acute toxoplasma infection during pregnancy can be prevented, its potentially tragic outcome for the fetus and newborn continue to occur in Iran (28). The infection can be acquired during ingestion through consuming undercooked meat or contaminated food or water. Previous studies indicated that the incidence and severity of congenital toxoplasmosis vary with the trimester during which the infection was acquired by the mother (20). Severe form of congenital toxoplasmosis is expected to be found in infants born of mothers who acquire toxoplasma infection in the 1st or 2nd trimester of gestation, whereas children born of mothers who acquire the infection during the 3rd trimester are mostly asymptomatic.

 Transmission to the fetus may be resulted in visual and hearing abnormalities, mental retardation, seizures, hematological disorders, or death (20, 27, 28). Congenital toxoplasmosis is complicated with encephalitis, microcephaly, hydrocephaly, hepatitis, lymphadenopathy and even intrauterine death. Presence of T.gondii in human placenta is associated with congenital infection (29). In a study conducted in Jahrom, South of Iran, by Davami et al., IgG-seropositivity among pregnant women measured by ELISA method was 13%, and for 2% IgM-seropositivity was (22).

In a study conducted in the north of Iran by Elahian et al., IgG-seropositivity among pregnant women referring to health center measured by ELISA method was 52%, no samples from them were positive for IgM-antibody reflecting no recent infection during gestation (28). In another study from Shiraz, a city in southern Iran, the authors concluded that toxoplasma might largely contribute to spontaneous abortion, due to high level of toxoplasma infection in aborted fetuses. In this study, Asgari et al found that 78 of 542 (14.4%) were positive for T.gondii using PCR method (29).

**Ocular toxoplasmosis:** *Toxoplasmosis *is one of the most common infectious cause of uveitis among immunocompetent individuals. Ocular lesions may result from both congenital and acquired infection, in which lesions may occur during the acute or latent stage of the infection (20). Although acquired *toxoplasmosis* in healthy adults is most commonly asymptomatic ocular toxoplasmosis in these individuals may end to complete or partial loss of vision. Patients with ocular toxoplasmosis who acquired utero infection are more frequent in the 2nd and 3rd decade of life and have more severe disease hallmarked by bilateral disease, old retinal scars, and involvement of the macula, whereas, patients in the setting of acute toxoplasmosis are more often between the 4th and 6th decades of life, most often have unilateral involvement, and have eye lesions that usually spare the macula and do not present with associated old scars (20).

In a study from southern Iran, which was conducted among new cases of uveitis, anterior uveitis was the most common type of inflammation (40%), followed by posterior uveitis (28%). Toxoplasmosis was reported as the most common cause of posterior uveitis (30). Toxoplasmosis as an important infective cause for anterior uveitis in Iranian patients is documented in other studies (31).

Symptoms of acute toxoplasmic chorioretinitis include blurred vision, scotoma, pain, photophobia, and epiphora. Loss of central vision happens when toxoplasmosis is associated with macula involvement. When inflammation resolves, vision improves, but without complete recovery of visual acuity (20).

**Diagnosis: **Since the clinical manifestations of *toxoplasmosis *are mostly nonspecific, toxoplasmosis should be carefully kept in mind in approaching a patient with clinical presentations suggestive of infection. The appropriate diagnostic tests must be done and correctly interpreted in regard to patient's clinical features.

There are many diagnostic methods for acute toxoplasma infection such as: isolation of *T. gondii *or amplification of its DNA in blood or body fluids; demonstration of tissue cysts tachyzoites in histopathology examination of tissue, cytological preparations of body fluids, histologic appearance of a biopsied lymph node, and serologic tests (20, 32-35). Here, because of resource limitation, technical laboratory facilities and hazards of spreading the infection in health network, we emphasize on serological tests for diagnosing toxoplasma infection in patients referring to health centers around the country. Toxoplasmic lymphadenitis in older children and adults are probably diagnosed based on histologic examination alone (33). Polymerase chain reaction (PCR) for the detection of *toxoplasma DND* in body fluids (e.g. Cerebrospinal fluid, blood, urine, amniotic fluid) and tissues (placenta, fetal) has been successfully used to diagnose congenital, ocular, cerebral, and disseminated toxoplasmosis. The sensitivity and specificity of PCR in CSF is 77% and 100%, respectively (19, 28, 31). In some other patients such as pneumonia or CNS infection, imaging must be added to diagnostic investigation (34).

**Serologic tests:** A large number of serologic tests are provided to detect specific antibody against T.gondii. There is no single serologic test to diagnose toxoplasma infection (20, 32).

**IgG-Antibodies Tests: **The most frequent used tests for IgG antibody detection are: Sabin-Feldman dye test, ELISA, indirect fluorescent antibody (IFA) test. Usually, IgG –Ab appears within 1 to 2 weeks after the initiation of toxoplasma infection, reaches to its peak within 1 to 2 months, then decreases slowly, and usually persists in low titers for life (20). Sabin-Feldman dye test because of living organism's requirement is only available a few reference laboratories. The diagnostic value of IFA test seems to be the same as the dye test (20). The agglutination test detects IgG antibody. The test is very sensitive to IgM antibody, and should not be used for the measurement of IgM antibodies.

The IgG-ELISA is now the most widely used test to detect IgG antibodies against T*. gondii.*

IgG- avidity test is a useful test to differentiate latest toxoplasma infection from distant infection. Low avidity test results may persist for a long time after the primary infection, hence a low avidity test result should not be used to diagnose recent infection. HCWs providing care of pregnant women should be aware that IgG-avidity tests are only confirmatory tests and should not be used alone to make a therapeutic decision (20).

**IgM- Antibodies Tests:** IgM antibodies appear earlier and decline more rapidly than IgG antibodies. IgM -Ab tests are very important as diagnostic methods to diagnose acute infection and to detect recent infection during gestation (20, 35). IgM-IFA-AB appears within the first week of infection; rapidly increases in titers, then titers decrease to a low level, and usually disappear few months later. Antinuclear antibodies and rheumatoid factor may cause false-positive results (20).

The double-sandwich IgM-ELISA is currently the most frequently used test for the detection of IgM antibodies against *T. gondii *in adults, fetuses, and newborns (20, 28, 32). *Immunoglobulin *M *Immunosorbent Agglutination Assay test:* IgM-ISAGA test is more sensitive and specific than the IgM-IFA test. The presence of RF or ANA does not cause false-positive results in the IgM-ISAGA. In adults, it is more sensitive but much less specific than the double-sandwich IgM-ELISA method. IgM-ISAGA is the most sensitive method for diagnosis of congenital infection in infants (20).

**IgA- Antibodies Tests:** Because of the increased sensitivity of IgA assays in comparison to IgM assays in the diagnosis of congenital toxoplasmosis, this test is considered by physicians in the serologic diagnosis of the infection in the fetus and newborn (20,32).

**Treatment: **Pyrimethamine is the most effective drug and should always be included in anti-toxoplasma drug regimens. Pyrimethamine as a folic acid antagonist may cause bone marrow suppression therefore; concomitant use of folinic acid (calcium leucovorin) is needed to decrease the drug side effects (20, 36). Since monotherapy is not recommended in the treatment of toxoplasmosis, a second drug such as sulfadiazine or clindamycin (37) should be added. The most frequent sulfadiazine side effects are skin rashes and nephrotoxicity. Sulfadiazine-induced side effects such as hallucinations, encephalopathy or a new psychiatric finding must be considered in an AIDS patient under treatment of toxoplasmosis. The most significant side effects of clindamycin are rash, nausea, vomiting, diarrhea (associated with *Clostridium difficile *infection) and less commonly myopathy (20).

Trimethoprim-sulfamethoxazole (TMP-SMX) as a folic acid antagonist drug combination is similar to pyrimethamine plus sulfadiazine, although limited it has documented activity against toxoplasmosis (6). Recent studies have suggested that other drugs including azithromycin, clarithromycin, atovaquone, and dapsone may have therapeutic effect on toxoplasmosis infection. Since this effect is less clear; they should only alternatively be used in combination with pyrimethamine whenever possible (20, 36). Although spiramycin is used to reduce transmitting the infection to the fetus in pregnant women, it has not been shown to be effective for toxoplasmosis treatment (32).

**Treatment of immunocompetent patients:** Treatment of immunocompetent patients with lymphadenitis is rarely indicated; this form is self-limited. Alavi et al., suggest that TMP-SMX (with a dose of 48 mg/kg/day divided into two doses for 1 month) has a good therapeutic results in children with lymphadenitis and can be administrated in selected patients for whom treatment is required (6). Treatment with pyrimethamine plus sulfadiazine was successful in a case of disseminated toxoplasmosis in an immunocompetent patient (24). 

**Treatment of Immunodeficient Patients:** Treatment of toxoplasmosis in AIDS patients as well other immunodeficient patients includes acute treatment, maintenance treatment, and prophylaxis. Pyrimethamine [oral 200 mg loading dose, then 50 (<60 kg) to 75 (>60 kg) mg PO qd] plus sulfadiazine [1000 (<60 kg) to 1500 mg (>60 kg) PO q6h] plus folinic acid [10 to 20 mg PO, IV, or 1M qd (up to 50 mg qd)] is the therapy of choice for AIDS patients with toxoplasmosis. Clindamycin with a dose of 600 mg q6h PO or IV (up to 1200 mg IV) q6h can be substituted with sulfadiazine. Possible alternative regimens may be as follows: TMP-SMX, 5 (as high as 15-20) mg/kg/ day (trimethoprim component) PO or IV q 12h. After successful primary therapy, drug doses are generally decreased for maintenance therapy; Pyrimethamine (25 mg/day) plus sulfadiazine (500 mg four times daily) (20).

**Treatment of ocular toxoplasmosis:** Ocular patients should be treated based on ophthalmologic evidence such as: decrease in visual acuity, macular or per papillary lesions, greater than one optic disc lesions, lesions associated with inflammation, multiple active lesions, and active disease for longer than I month, and any ocular lesions in association with acquired infection. The classic therapy for ocular toxoplasmosis includes a combination of pyrimethamine (100 mg loading dose given over 24 hours, followed by 25 to 50 mg/day) and sulfadiazine (1 g given four times daily) for 4 to 6 weeks depending on the clinical response (20).

TMP-SMX (TMP, 48 mg/kg/day divided into two doses) or clindamycin (300 mg PO every 6 hours for a minimum of 3 weeks) has also been associated with acceptable results (20).

**Treatment of pregnant women and infected neonates:** In pregnant woman suspected to have active toxoplasmosis infection or recent infection during gestation, prophylaxis with spiramycin begins (32). Sulfadiazine or clindamycin alone may be used if spiramycin cannot be used or is not available (20). The treatment is changed to pyrimethamine plus sulfonamide plus folinic acid if fetal infection is proven. Confirmed infected children are treated with sulfadiazine (50 mg/kg every 12 hours), pyrimethamine (loading dose: I mg/kg every 12 hours for 2 days; then beginning on day 3, 1 mg/kg/day for 2 or 6 months; then this dose three times weekly and folinic acid (10 mg three times weekly )for 12 to 24 months (20,32). All the children from mother infected during gestation are followed-up after birth to detect the consequence of toxoplasma infection, mainly ocular (38). 

**Prevention: **Seronegative pregnant women and immunodeficient patients are the most important population and should exeruse caution to avoid the risk of getting active infection. Prevention is readily accomplished by health education and increasing the knowledge of these patients about toxoplasmosis, its routes of transmission and ways of decreasing its complications. The goal is to avoid the ingestion of and contact with tissue cysts or speculated oocytes (20, 39). 

Serological screening of donors and recipients before organ transplantation is the most important diagnostic method to identify high risk patients (seropositive recipients and seropositive donor/seronegative recipients) for toxoplasmosis. Preventing toxoplasmosis in immunodeficient patients relies on chemoprophylaxis with TMP/SMX (40). The most important recommendation to prevent and spread toxoplasma infection through the community especially among pregnant women and immunodeficient patients are as follows below:

*Reduction of environmental contamination: by *reducing the risk of spread of the infection via environmental factors through preventing the food and water from being contaminated with T.gondii. Reduce the risk of cat induced environmental contamination by feeding cats with safe food including cooked meats, disinfecting the cat-related material and holding place (19). *Reducte transmission to people: R*educe toxoplasma transmission to people by avoiding direct handling of stray cats, especially kittens, and avoiding contact with soil.

The measures to reduce transmission of toxoplasmosis through food or water, including eating the previously frozen meat; treating drinking water to water, cooking food with safe water; careful washing of dishes, counters, and utensils, and appropriate washing hands with water ad soap after contact with raw meat, poultry, seafood, or unwashed fruits or vegetables (41).

**Screening high risk population:** Pregnant women should be screened to detect infection during pregnancy and appropriate treatment (32). Screening of immunodeficient patients such as AIDS and donors and recipients before organ transplantation and administering TMP/SMX (40).

In conclusion, this review article showed that Iranian Health Care Network has enough capacity to integrate anti toxoplasma activities throughout the country from the first line health unit, rural health centers, and urban health centers to tertiary hospitals. The findings of the present study showed a practical view about epidemiology of toxoplasma in Iran and other parts of the world. The current review using updated data including more recent data from the recently published journals corroborated the findings from the previous studies (5-8, 13, 17-20, 23, 26, 35). This review showed that previous ideas about toxoplasmosis regarding to prevalence, clinical presentation among immunocompetent individuals, relation to non-infectious diseases such as schizophrenia, polymiositis, fever of unknown origin in immunocompetent hosts are changing. 

As mentioned before, this study, however, showed some beneficial new ideas for diagnostic methods based on availabilities in limited resource area in rural and even remote urban health centers in Iran. This finding is of high importance, because this information enables general physician to choose proper laboratory tests and appropriate drugs based on their availabilities in health settings. The present study showed that nontoxic and available drugs such as co trimoxazole and clindamycin may be used instead of toxic and non-available drugs such as pyrimethamine and sulfadiazine (6, 37). 

The current review also included data about toxoplasmosis in pregnant women, HIV infected patients, ocular toxoplasmosis and strategies for the prevention of toxoplasma infection. Collecting a useful data in a single article based on epidemiological pattern of toxoplasma infection in Iran according health facilities in the region enables general physicians to approach and manage their patients with toxoplasmosis better than the past.

Pregnant women and immunodeficient patients should be screened to detect infection. IgM -Ab tests are very important used diagnostic methods to diagnose acute infection and to detect recent infection during gestation. HCWs providing care to pregnant women should be aware that IgG-avidity tests are only confirmatory tests and should not be used alone to make a therapeutic decision. Treatment with pyrimethamine plus sulfadiazine plus folinic acid is the first choice treatment regimen in an immunocompetent patient. Health education including recommendation to prevent toxoplasma infection in pregnant women and immunodeficient patients should be provided.

## Notes

**Funding: **This study has been funded by the infectious and Tropical Research Center of Joundishapur University of Medical sciences; and no financial support from other institutes.
